# Interactions between O_2_ Nanobubbles and the Pulmonary Surfactant in the Presence of Inhalation Medicines

**DOI:** 10.3390/ma15186353

**Published:** 2022-09-13

**Authors:** Katarzyna Dobrowolska, Marcin Odziomek, Karol Ulatowski, Weronika Kędziora, Karolina Soszyńska, Paweł Sobieszuk, Tomasz R. Sosnowski

**Affiliations:** Faculty of Chemical and Process Engineering, Warsaw University of Technology, Waryńskiego 1, 00-645 Warsaw, Poland

**Keywords:** interfacial rheology, dispersion of oxygen nanobubbles, pulmonary surfactant, air–liquid interface

## Abstract

A dispersion of oxygen nanobubbles (O_2_-NBs) is an extraordinary gas–liquid colloidal system where spherical gas elements can be considered oxygen transport agents. Its conversion into inhalation aerosol by atomization with the use of nebulizers, while maintaining the properties of the dispersion, gives new opportunities for its applications and may be attractive as a new concept in treating lung diseases. The screening of O_2_-NBs interactions with lung fluids is particularly needed in view of an O_2_-NBs application as a promising aerosol drug carrier with the additional function of oxygen supplementation. The aim of the presented studies was to investigate the influence of O_2_-NBs dispersion combined with the selected inhalation drugs on the surface properties of two types of pulmonary surfactant models (lipid and lipid–protein model). The characteristics of the air–liquid interface were carried out under breathing-like conditions using two selected tensiometer systems: Langmuir–Wilhelmy trough and the oscillating droplet tensiometer. The results indicate that the presence of NBs has a minor effect on the dynamic characteristics of the air–liquid interface, which is the desired effect in the context of a potential use in inhalation therapies.

## 1. Introduction

Nanobubbles (NBs) are gas domains with a boundary diameter still broadly discussed; however, in many sources, their upper diameter is limited to 1 µm, as this is a boundary of rapid change in gas bubbles properties [1,2]. Such size ensures a huge surface-to-volume ratio and possibly very high internal gas pressure. Due to the low buoyancy compared to viscous forces and diffusional motions, nanobubbles’ rising velocity is low. Combined with the still discussed and not fully understood stabilizing mechanisms, it contributes to the excellent stability of NBs in liquids [3,4]. Their liquid dispersions are of interest in the various fields of advanced science, technology, and medicine due to an immense potential for improving the diffusion and transport processes of gases [5,6,7,8,9]. Many works report that treatment with water saturated with oxygen nanobubbles results in the enhanced proliferation of cells and even whole-organism growth [10,11,12]. Another important ability determining the usefulness of NBs in some areas is the generation of free radicals such as hydroxyl radicals (·OH) in the presence of NBs. Especially, ozone NBs demonstrate oxidizing power sufficient enough even for the decomposition of some pollutants that are not easily decomposed under normal conditions (e.g., organic phenols) [5,8,9]. On the one hand, NBs which are able to generate a high concentration of reactive oxygen species (ROS) are preferable for usage in some applications, such as wastewater treatment, but on the other hand, for medical applications high number of ROS, such as hydroxyl radicals, may not be welcome.

NBs that have found application in medicine are generated mainly from oxygen or nitrogen as they have much lower oxidative potential than ozone NBs and can be used in contact with living matter [10,11,12,13,14,15].

One of the most prominent branches of medicine which takes advantage of nanobubble addition is the treatment of ischemic foot wounds using the Carbothera device [16,17]. Other medical research directions involving nanobubbles are target therapies for tumors, ultrasound imaging, and plasmid DNA delivery, among others [14,18,19,20]. Nanobubbles are also used as drug carriers for drug delivery [21], mainly in an encapsulated form with a stabilizing shell comprising polymers, phospholipids, or proteins. We propose to use the free O_2_-NBs that are formed in the liquid phase without a shell in the role of a new solvent and carrier for inhalable medicines delivered by nebulization. Generally, aerosol therapy constitutes one of the most efficient routes of drug administration to the organism. Even though it allows for the effective treatment of both systemic and pulmonary diseases, its prominent use is in treating respiratory disorders. As many of them are related to impaired gas exchange, the successful application of O_2_-NBs dispersions as an additional source of oxygen to the respiratory system could enhance the therapeutic effect of inhalation, which seems particularly important in the face of the ongoing SARS-CoV-2 pandemic and its severe consequences such as post-acute COVID-19 syndrome [5]. Lung damage in the course of COVID-19, but also many other diseases, often leads to acute hypoxic respiratory failure and may eventually lead to acute respiratory distress syndrome (ARDS). The main therapeutic goal in such a situation is to maintain gas exchange at an appropriate level and prevent the intensification of changes in the lung parenchyma. Depending on the severity of hypoxemia, different techniques can be used to improve oxygenation. In some patients, conventional (passive) oxygen therapy alone is sufficient; however, in patients with worsening respiratory failure, high flow nasal oxygen therapy or even invasive ventilation must be used [22]. What is most important, oxygen therapy is often accompanied by delivering various medications through inhalation (antibiotics, corticosteroids), mostly using nebulizers. Using liquid dispersions of O_2_-NBs as a solvent for a medicine administered by nebulization would potentially have an additional advantage of introducing simultaneously much more oxygen to the surface of the respiratory tract.

Our previous studies [23] showed that aqueous dispersions of oxygen nanobubbles fulfill the stability requirements during storage. Furthermore, their potential application does not require changes in the construction of the main types of nebulizers present in the market. What is most important is that the increased oxygen content in the aerosol generated from O_2_-NBs dispersions in the vibrating mesh nebulizers suggests that the proposed concept may be considered a potential additional source of oxygen delivered to the respiratory tract during inhalation treatment.

However, administering medicines by inhalation meets various challenges regarding safety that must be considered when developing new formulations and products. For example, it demands overcoming some specific mechanisms protecting the respiratory tract from the harmful influence of pollutants and pathogens present in inhaled air with simultaneous preservation of the delicate chemical composition of the liquid layer covering the surface of the respiratory tract. Whereas the mucus present in the upper parts of the bronchial tree plays an essential role in mucociliary clearance functioning [24,25], the pulmonary surfactant (PS) is responsible for preserving breathing mechanics and the clearance processes in the alveoli [26,27]. Generally, the PS is a complex mixture of phospholipids and proteins synthesized and secreted by alveolar type II epithelial cells into the alveoli that creates a unique interface separating alveolar gas and liquids at the alveolar cell surface, reducing surface tension and maintaining lung volumes at the end-expiration [28,29]. Since the surfactant plays an essential role in the mechanics of the respiratory system, a serious disturbance of its properties will consequently lead to breathing problems, even as critical as ARDS [30]. The surfactant can be inactivated at various points in its “life cycle”—in short, from transcription and translation of proteins, through secretion into the hypophase liquid layer, transport through subphase to the alveolar interface, or losses due to transport out of the alveoli to the airways [28]. One of our previous in vitro experiments [30] also clearly indicated a possibility of PS deactivation by different toxic agents, such as acidic substances inhaled with air or exposure to the air containing neutral alkali–acidic gas, namely ozone. In such a case, perhaps some free radical reactions may be important. Due to the possibility of creating free radicals in the dispersions of oxygen nanobubbles, it is necessary to investigate their eventual interactions with pulmonary surfactants. It will help to assess the safety of the proposed conception.

Nanobubbles display unexpected usability in various branches of science and industry. We decided to check if there is a possibility of using them in aerosol therapy. The results of this work are a continuation of our research on the influence of oxygen nanobubbles on the quality of aerosols released from medical nebulizers [23]. These earlier results demonstrated that the atomization of O_2_-NBs dispersions in selected nebulizers does not change the aerosol quality, so the expected deposition of the inhaled droplets in the lungs is unchanged. Moreover, the increased oxygen content in the aerosol was maintained, indicating the potential usefulness of the proposed by us solution. As previous work was carried out in the water and the final aim is to supply nanobubble-carried drugs to the pulmonary system, this work broadens the investigations of the surface properties of pulmonary surfactant models to mixtures of O_2_-NBs with salt and selected inhalation drugs. Additionally, the experiments were conducted under conditions of physiological temperature that, due to complexifying, are not typically considered by other researchers [31,32]. Efforts were made to find an answer regarding the influence of liquid dispersions of O_2_-NBs on the activity of pulmonary surfactant in conditions close to physiological and, in consequence, the safety of the proposed conception.

## 2. Materials and Methods

### 2.1. Generation of O_2_-NBs Dispersions in Water (ADON—Aquous Disspersion of Oxygen Nanobubbles) and Saline (SDON—Saline Disspersion of Oxygen Nanobubbles)

The liquid dispersions of O_2_-NBs were prepared according to the procedure presented in detail by [23]. In brief, oxygen nanobubble dispersions in water (ADON) or 0.9% saline (SDON) were obtained using the designed generation setup equipped with a cylindrical porous flat ceramic (ZrO_2_ on TiO_2_ support) membrane (pore diameter 0.14 μm, internal/external membrane diameter: 8/10 mm, membrane length 125 mm; Tami Industries, France). NB formation took place during a 30-min process at controlled oxygen pressure and optimized volumetric flow rates of liquid and gas. The ADON and SDON samples were stored in glass vials that were closed and secured with parafilm. Prior to the studies with the pulmonary surfactant models, the properties of the liquid dispersions of O_2_-NBs were determined in terms of NBs size distribution and oxygen content [23].

### 2.2. Experimental Evaluation of Interactions between Oxygen Nanobubbles and Pulmonary Surfactant Models

In vitro studies on the influence of the O_2_-NBs dispersions on two models of the pulmonary surfactant were carried out using Langmuir–Wilhelmy balance and pendant drop tensiometry. Each method demands using a suitable model of the PS: the two-component lipid model (LM) and a complete model of the pulmonary surfactant (MPS) containing lipids and the proteins occurring naturally in the lungs, respectively.

#### 2.2.1. Interactions with the Lipid Model (LM) of the Pulmonary Surfactant

1,2-Dipalmitoyl-sn-glycero-3-phosphocholine (DPPC, synthetic, 99%, Lipoid GmbH, Ludwigshafen, Germany) and cholesterol (CHOL, 99%, Sigma-Aldrich/Merck, Kenilworth, NJ, USA) were used without additional purification. DPPC and CHOL were mixed at a molar ratio of 8:2 and dissolved in chloroform (Merck, Kenilworth, NJ, USA). The prepared solution of lipids was added dropwise with a Hamilton microsyringe onto the water subphase (ultrapure water obtained from water purification system Direct-Q—Merck, Kenilworth, NJ, USA) in the Langmuir trough model: KSV NIMA (dimensions: 364 × 75 × 4 mm—Biolin Scientific, Espoo, Finland) placed on an anti-vibration table. After spreading, the lipid monolayer was left for a couple of minutes for solvent evaporation and equilibration.

The reference data were obtained for DPPC and DDPC+CHOL monolayers on pure water.

To study the effects caused by oxygen nanobubbles, aqueous dispersions (ADON) or dispersions in physiological saline (SDON) were added to the interface so that their final concentration in the system was 0.018 mL/mL. This value was adapted from the previous studies as a result of the predicted deposition of the ADON or SDON aerosols delivered by inhalation [23]. Additionally, the possibility of using O_2_-NB dispersion as an alternative carrier for the selected drugs was investigated by preparing the ADON or SDON mixtures (1/1 vol/vol) with the nebulization medicines of ectoine (Ectodose, Solinea, Ciecierzyn, Poland) or budesonide (Budixon-NEB 0.5 mg/mL, Adamed, Pieńków, Poland). The prepared liquid mixtures were applied to the interface in a similar way as ADON or SDON without drugs. Ectoine (E) is a substance known for protective properties that minimize protein denaturation by the removal of water molecules [33], and it is used in inhalation therapies supportive in allergy treatment. Budesonide (B) is a glucocorticosteroid characterized by a strong affinity for receptors located in the bronchi. It has a diastolic effect, which is particularly desirable during a strong allergic reaction and in the treatment of diseases such as COPD [34]. Due to the insolubility of budesonide in water, inhalation medicines containing budesonide are formulated as aqueous microsupensions stabilized by small amounts of Tween 80 and salts.

The measurements of surface pressure (π) in the Langmuir trough during the symmetrical compression of the interface (barrier speed of 40 mm/min) were conducted using filter paper (Whatman) as the Wilhelmy plate. All of the measurements were carried out at 36.6 ± 0.1 °C. The determined π-A isotherms were used to calculate the surface compressibility factor (c_s_^−1^) according to the formula:

(1) $$c_{s}^{-1}=-A\frac{d\pi }{\mathrm{dA}}\;$$

where π is defined as the difference between the surface tension of the subphase (σ_sub_) and the instantaneous surface tension in the system (σ), and A is the interfacial area. This factor reflects the elasticity of the interfacial film during compression, and Equation (1) can be also used as the definition of Gibbs elasticity [35,36].

Based on the analyzed compressibility factor c_s_^−1^(π) and using the Davies and Rideal criteria [37], the thermodynamic states of the monolayer were determined. According to the adopted criteria, the compressibility factor is in the range of 12.5–100 mN/m for the expanded liquid state (LE), 100–250 mN/m for a condensed liquid state (LC), and above 250 mN/m when the monolayer state is identified as solid (S).

#### 2.2.2. Interactions with the Multicomponent Pulmonary Surfactant Model MPS (Lipids + Proteins)

Calfactant—a sterile pulmonary surfactant suspension obtained from calf lungs (Infasurf, ONY Biotech, Amherst, NY, USA)—was used as a multi-component model of pulmonary surfactant, MPS. This model has an advantage over LM since it contains a mixture of lipids (phospholipids and neutral lipids) and hydrophobic surfactant-associated proteins (SP-B and SP-C), which means that it is closer to a physiological system. Infasurf was diluted with water to a phospholipid concentration of 5 mg/mL and then mixed with ADON or SDON to obtain their final concentration of 0.018 mL/mL. In addition to ADON and SDON added to the MPS, their mixtures with ectoine or budesonide medicines were also prepared and used, as described earlier.

The influence of NBs on MPS was studied using the pendant drop tensiometer PAT-1M (Sinterface, Berlin, Germany) under thermostatic conditions (36.6 ± 0.3 °C). The measurements of dynamic surface tension were carried out during sinusoidal drop oscillation at frequencies of f = 0.1, 0.125, 0.25, 0.33, and 0.5 Hz, which were selected as being comparable to the frequencies of alveolar pulsations during human breathing at various states of physical activity. At the beginning of each test, the freshly formed air–liquid interface was left until reaching the equilibrium and next forced to oscillate with 10% amplitude of the interfacial area variations.

**Figure 1:**
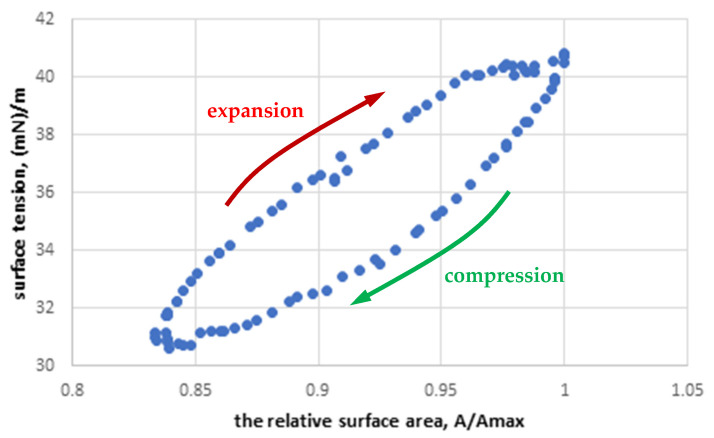
Exemplary surface tension hysteresis obtained experimentally for MPS (2.5 mg/mL) at f = 0.1 Hz.

Based on the obtained data, the effective dilatational rheological parameters of the air–liquid interface, i.e., the surface dilatational elasticity (ε) and surface dilatational viscosity (µ) were determined. Both parameters indirectly illustrate the alterations in the surface tension hysteresis (Figure 1) that results from mechanical properties of the interface and/or mass exchange between the interfacial region and the subphase.

## 3. Results and Discussion

### 3.1. Characterization of Two-Component Monolayers (LM)

The study of the air/liquid interface, containing phospholipids and neutral lipid characteristics for pulmonary fluid, was started by investigating the isothermal compression of the monolayers. In consequence, we have obtained the isotherms of surface pressure π(A) (data shown in the Supplementary Material), allowing us to determine the compressibility factor (Equation (1)).

**Figure 2:**
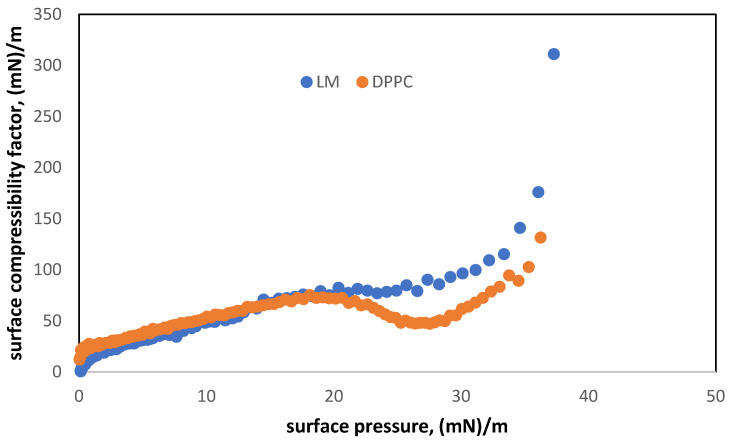
Isothermal surface compressibility factor of DPPC and mixed LM (DPPC + cholesterol) monolayer on the aqueous subphase.

The relationship of the compressibility factor on the surface pressure (Figure 2) shows the changes in the organization of DPPC molecules interacting with cholesterol molecules on the air/water interface. The presence of cholesterol is responsible for smoother transitions between the states of the monolayer. Moreover, the cholesterol molecules spread in the lipid monolayer form a more tightly packed layer than in the case of pure DPPC through a sudden increase in surface pressure, which has been described in the literature [38,39] as the typical behavior of highly rigid monolayers.

We can observe the LC state in the small range of the surface pressure (about 31.2–37 mN/m) and an expanded liquid state in the wide range (4.0–31.2 mN/m). Below 4 mN/m, the monolayer is in a gaseous state. A difference between the compressibility factor of both monolayers (DPPC and DDPC + CHOL) can be found in the surface pressure range of 20–35 mN/m, where c_s_^−1^ for DPPC shows a visible minimum (at 25–27 mN/m). It is associated with the coexistence region of LE and LC phases which disappears in the mixed DPPC/cholesterol monolayer [38].

These data show that even if DPPC is most often used as the simplest model of PS [30,39,40], the addition of CHOL notably changes the characteristics of the lipid monolayer, allowing a more realistic representation of the natural system [41].

### 3.2. Interactions between Oxygen Nanobubbles and the Lipid Model (LM) of the Pulmonary Surfactant

**Figure 3:**
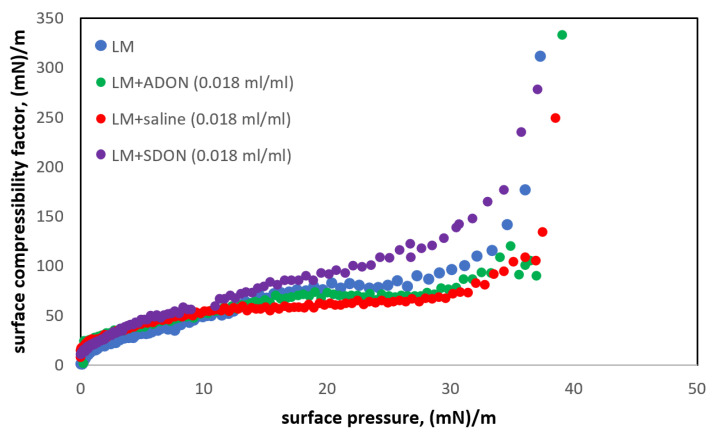
Isothermal surface compressibility factor of LM monolayer on the aqueous subphase in the presence of ADON or SDON.

Figure 3 shows the effect of oxygen nanobubbles on the LM. In addition to ADON (O_2_-NBs in water), the NB dispersion in physiological saline (SDON) was considered a more desirable carrier in medical inhalations (saline preserves the osmotic balance), although the presence of salt may adversely affect the stability of oxygen nanobubbles [3,42]. The mechanisms governing this phenomenon are still not fully understood, but it is known that NBs’ stability also depends on the storage method of the dispersion [3,23].

The presence of oxygen nanobubbles in the subphase and possible NBs adsorption to the lipid monolayer on the air–liquid interface can influence its properties and stability, which is important in the view of PS properties and functions. Analyzing the values of the compressibility factor and, thus, the transitions between monolayer states, it is found that the presence of NBs has only a minor influence in the surface pressure range below 10–12 mN/m (Figure 3). However, there is a visible difference in the c_s_^−1^ value for π above 15 mN/m. The compressibility factor for LM in the presence of ADON is similar to LM on saline but lower than for LM on water or with SDON. It suggests that ions slightly reduce c_s_^−1^, but the trend is changed when oxygen NBs and saline act together. Since in all cases there is a visible LC or even S state, this allows us to conclude that oxygen NBs do not affect the LM dynamics in the surface pressure range that are physiologically relevant (above 30 mN/m, i.e., when surface tension in the system is lower than 40 mN/m).

**Figure 4:**
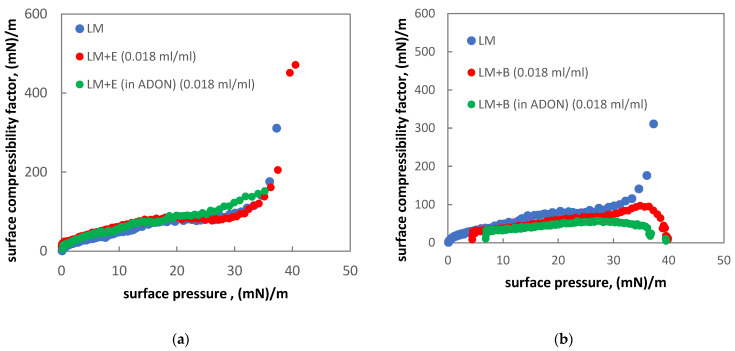
Isothermal surface compressibility factor of LM monolayer on the aqueous subphase: (**a**) in the presence of ectoine pharmaceutical solution (E) diluted with water or ADON, (**b**) in the presence of pharmaceutical drug with budesonide (B) diluted with water or ADON.

Figure 4a,b show the effect of drugs and their mixtures with ADON/SDON on LM. There is almost no change in c_s_^−1^ of LM if ADON is mixed with ectoine (Figure 4a). In contrast to the results obtained for pure ADON/SDON and ADON with ectoine, the strong effects in the surface pressure above 30 mN/m can be detected for budesonide, both without and with oxygen NBs present in the system (Figure 4b). The influence of budesonide medicine on the monolayer may be caused by two factors. Firstly, it is a suspension that contains insoluble submicrometer-size particles of the steroid, which may be incorporated into the hydrophobic part of the monolayer. Secondly, the medicinal product contains surface-active additives, which adsorb at the air–liquid interface. Both factors influence the compression isotherm π(A), resulting in a significant change in the c_s_^−1^ (π) relationship. O_2_-NBs (ADON) in the mixture with budesonide cause an additional reduction in the compressibility factor (Figure 4b).

These findings partially agree with our previous results on budesonide interactions with the pulmonary surfactant [43]. It can be seen that NBs with budesonide reduce the elasticity of the LM monolayer at a physiologically relevant range of the surface pressures (i.e., above 30 mN/m), which indicates the inability of the formation of a tightly-packed monolayer. It should be noted, though, that the presented data have been obtained in a simplistic model (LM) and at much slower surface compression than in the real PS system, which sets the limitations of the physiological significance of these results.

### 3.3. Dynamics of Air–Liquid Interface of Multicomponent Pulmonary Surfactant Model MPS (Lipids + Proteins)

The characterization of the air/liquid interface containing the lipid–protein mixture of MPS was started by studying the time-dependent decrease in the surface tension at a constant interfacial area due to surfactant adsorption (data shown in the Supplementary Material). The mean value of the quasi-equilibrium surface tension after 10-min adsorption was 32.07 mN/m, which is comparable to the value expected in the lungs in vivo at rest [44].

**Figure 5:**
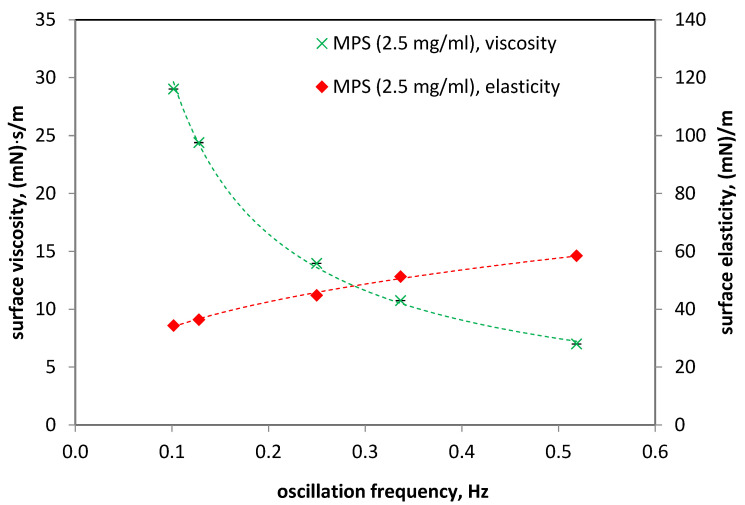
The effective dilatational rheological parameters of air–liquid interface with the lipid–protein model of the pulmonary surfactant, MPS (concentration 2.5 mg/mL).

The dynamic characteristics of the MPS interface during oscillation around this equilibrium value allowed us to determine the dilatational surface viscosity and elasticity as a function of oscillation frequency (Figure 5). These relationships are typical for surface active materials, and the pulmonary surfactant studied in vitro [45]. Higher oscillation frequencies result in a decrease in surface viscosity and an increase in elasticity. The values of surface viscosity change from 29 mN·s/m at the lowest deformation rate (f = 0.1 Hz) to 7 mN∙s/m at an oscillation frequency of 0.5 Hz. Simultaneously, the value of surface elasticity increases from 34 mN/m to 58 mN/m for the same frequency range. The observed relationships confirm the viscoelastic nature of the interface, and, at the same time, explain the occurrence of the surface tension hysteresis phenomeon in the MPS system (shown in Figure 1) and the frequency-dependent alterations of the hysteresis shape. A convenient parameter illustrating the dynamics of changes in the shape of the surface tension hysteresis is the phase (or: loss) angle, φ. Based on the viscoelastic model of the interface, it can be shown that the phase angle is defined as [46]:

(2) $$\varphi =\arctan\frac{\omega \mu }{\varepsilon }$$

where the oscillation rate, ω:

(3) ω=2πf

For the increasing deformation rate (i.e., higher oscillation frequency), a decrease in φ is expected as the viscosity nonlinearly decreases with the frequency (Figure 5). It means that the compression and expansion branches of the hysteresis curve will approach each other, indicating that the elastic component of the interface becomes dominant over the viscous one.

### 3.4. Influence of Oxygen Nanobubbles on MPS

**Figure 6:**
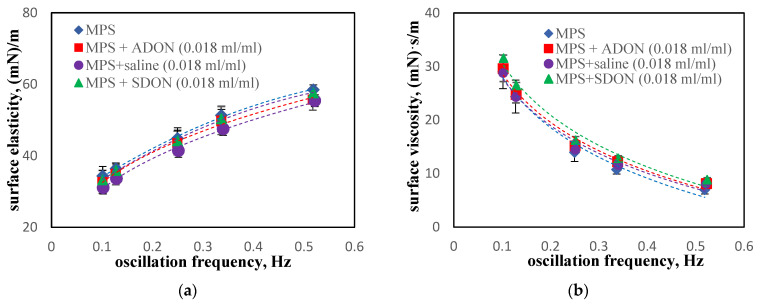
Dilatational surface elasticity (**a**) and viscosity (**b**) of air–water or air–saline interface in presence of MPS and ADON or SDON.

The effective rheological parameters in the MPS system after adding O_2_-NBs dispersions in water or saline follow the trends observed for pure MPS previously shown in Figure 5. There are no visible changes in the surface viscosity and elasticity vs. oscillation frequency, which suggests that neither ADON nor SDON influence MPS dynamics during surface deformations (Figure 6).

**Figure 7:**
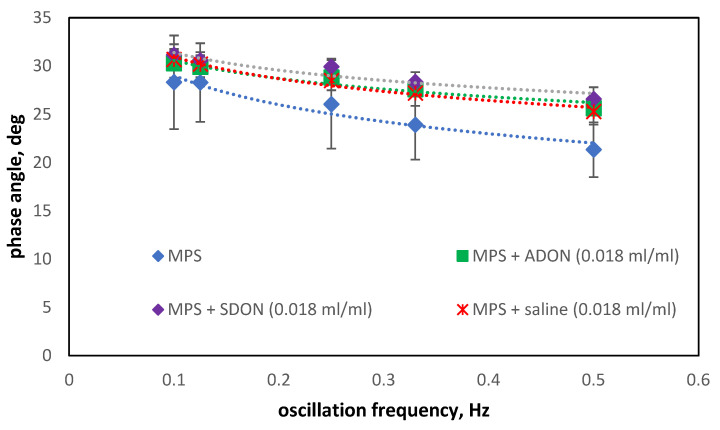
Changes of the phase angle in the oscillation of air/liquid interface with MPS (concentration of 2.5 mg/mL) in presence of saline, ADON or SDON.

The constant viscous properties of the interface suggest the absence of additional intermolecular interactions related to, e.g., the formation of H-bonds between the surfactant adsorbed on the surface and the water molecules [47]. Analyzing the changes in phase angle values in the presence of ADON and SDON (Figure 7), it can be noticed that tendencies of decreasing φ (hysteresis tapering) with increasing oscillation frequency are maintained. Moreover, the presence of salt ions and NBs only slightly increases the hysteresis width. A wider hysteresis loop denotes a larger amount of surface energy lost by the system, but it was proposed that the energy is not dissipated but converted to interfacial convection, which facilitates the mass transfer, and therefore the hysteresis is recognized as beneficial for the physiological functions of PS [26,27]. As seen in Figure 7, the differences between the phase angle values in the MPS + saline, MPS + ADON, and MPS + SDON systems are negligible. It shows that the addition of ADON can substitute saline in that manner. These results show that O_2_-NBs do not cause undesired effects in MPS characteristics during the breathing-like oscillations of the air–liquid interface.

### 3.5. Influence of Drugs and Oxygen Nanobubbles on MPS

**Figure 8:**
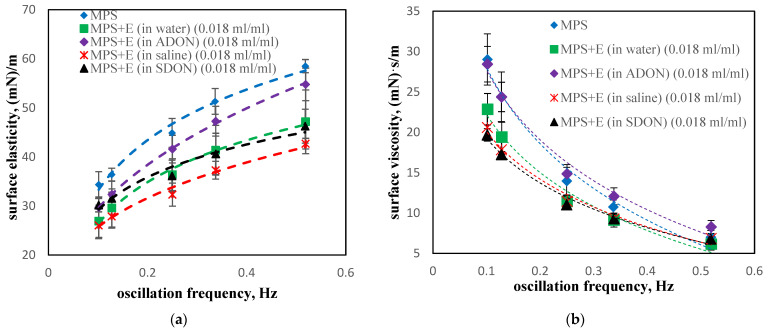
Dilatational surface elasticity (**a**) and viscosity (**b**) of MPS in the presence of the drug with ectoine (E) diluted in water, saline, ADON, or SDON.

Changes in the surface rheological properties of the air–liquid interface of MPS in the presence of drugs (ectoine or budesonide) and oxygen NBs, are different than in the case of pure ADON and SDON discussed earlier. The addition of ectoine (E—Figure 8) in water or saline reduces the surface elasticity indicating MPS interactions with the drug. The influence of electrolytes (black triangles in Figure 8a) is small compared to pure water (green squares). However, ectoine with NBs in water (denoted as E in ADON) leads to higher values of surface elasticity than in saline (E in SDON), which suggests synergic interactions between NBs and electrolytes with MPS, similarly as shown earlier for SDON without E. This conclusion is supported by μ(f) relationships, which show similar trends (Figure 8b).

**Figure 9:**
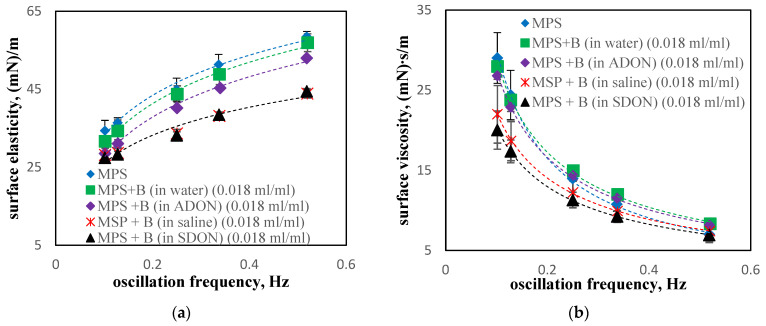
Dilatational surface elasticity (**a**) and viscosity (**b**) in the presence of the drug containing budesonide (B) diluted in water, saline, ADON, or SDON.

Similar tendencies to those observed in Figure 8 are noticed for budesonide, B (Figure 9). The addition of budesonide drug in water as well as in saline reduces the dilatational elasticity of MPS. The presence of NBs (in ADON/SDON—violet and black markers in Figure 9a) has no significant influence on the elasticity values compared to MPS + B (in water or saline—green and red markers). These results clearly show that changes in the dilatational rheological parameters are caused by the drug formulation rather than by NBs.

The surface viscosity of MPS is only slightly changed at f > 0.25 Hz; however it is decreased by budesonide medicine (in various solvents) at f = 0.1–0.15 Hz (Figure 9b). Similar to elasticity, the effect of oxygen NBs is small compared to the effect caused by the drug. The presence of NaCl in the solvent (physiological saline or SDON) results in the lowest values of surface viscosity, which confirms the role of electrolytes and the synergic effects of NBs and ions.

**Figure 10:**
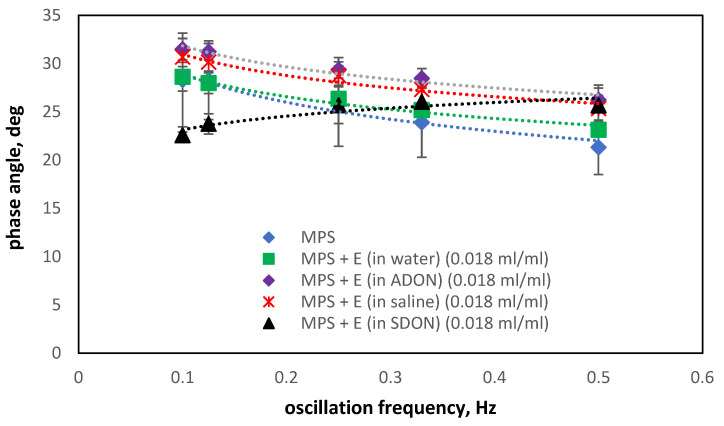
Changes of the phase angle in the oscillation of air/liquid interface with MPS (concentration of 2.5 mg/mL) in presence of ADON or SDON and ectoine (E).

**Figure 11:**
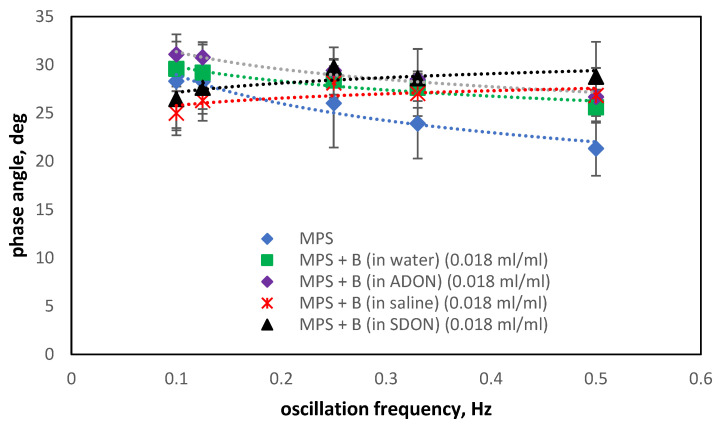
Changes of the phase angle in the oscillation of air/liquid interface with MPS (concentration of 2.5 mg/mL) in presence of ADON or SDON and budesonide (B).

Analyzing the changes in the phase angle values in the presence of ectoine (Figure 10) and budesonide (Figure 11), it can be found that the typical decrease φ with increasing oscillation frequency is maintained but not for systems where both saline and O_2_-NBs are present in the system. The presence of drug products without NBs (green squares in Figure 10 and Figure 11) always reduce the width of the hysteresis (φ is decreased).

The addition of NBs in water (ADON—violet diamonds) suggests a slight increase in hysteresis. This trend is altered in the presence of sodium and chloride ions (SDON), where the increase in f results in the increase of φ (black triangles). Again, we can attribute these changes to the synergic interactions of the surface-active additives of the drug product and the strong electrolyte forming the surface charge of the monolayer. The effects are amplified by oxygen nanobubbles; however, the variations in the phase angle are small (range of 20–32 deg). As the effects of the sole drug addition and the effects of saline itself are different from the effect of the addition of these substances together, we can see some kind of synergy between them, which provides us with these interesting results. These results are very promising regarding the safety of the O_2_-NBs dispersions for the pulmonary surfactant functions in vivo.

Summarizing the presented results, we can state that:

1. The selection of the PS model for the studies is important. The LM model is simpler and allows us to evaluate the changes in the surface processes more precisely using a Langmuir trough; however, it does not inform about the full dynamics of the PS system due to poor reconstruction of the composition of the interfacial layer and low deformation (compression) rates compared to the physiological system. The MPS model and dynamic surface oscillations in the pendant drop method reflect the dynamics of the physiological system and provide quantitative results reflecting the surface response to sinusoidal deformation.
2. The influence of oxygen nanobubbles on both models of PS is marginal. The PS properties are more altered by the drug product components (e.g., surfactants present in budesonide suspensions) than by O_2_-NBs. Since the safety of tested inhalation drugs (ectoine and budesonide) on the pulmonary system has been confirmed in clinical studies, it seems that very small effects on the PS surface activity detected here for NBs present no issue regarding the safety of their future use as drug carriers in medical inhalants.

## 4. Conclusions

The possible future application of oxygen nanobubbles in medical inhalations should consider their safety for the respiratory system, so the current work was focused on the measurements of surface interactions between nanobubble dispersions and the pulmonary surfactant.

We presented the results of the experiments on surface interactions between O_2_-NBs dispersions (water—ADON; saline—SDON) and different pulmonary surfactant models: the two-component lipid model (LM—studied in the Langmuir trough) and multi-component model (MPS—studied in the oscillating drop tensiometer). After characterization of both surfactant models in physiological temperature, we showed the influence of nanobubbles (ADON/SDON) in the presence of selected drugs delivered by inhalation: ectoine (E) and budesonide (B).

The study assessed the qualitative and quantitative influence of NBs nanostructures on the properties of pulmonary surfactant, using a number of parameters such as equilibrium surface tension, compressibility coefficient, surface elasticity, and surface viscosity. The results showed that the effect of O_2_-NBs on the surface properties of the PS models is small compared to the effects caused by the solvent (i.e., saline or diluted drugs). Additionally, some synergic effects of dissolved NaCl and oxygen NBs were observed, suggesting the role of electrolytes in NBs interaction with the pulmonary surfactant components.

The results allow us to conclude that oxygen nanobubbles are generally safe regarding their direct physicochemical influence on the pulmonary surfactant, so they have a practical potential as an additional component of inhaled aerosol droplets that should help to increase the oxygen transport in the lungs. However, it is reasonable to lead further research focused on the interactions of O_2_-NBs with other components responsible for the proper functioning of the respiratory tract, i.e., bronchial mucus. The results obtained in this study also constitute the key step for future research concerning the hydrodynamics and modeling of the upper respiratory tract with nanobubbles present in the pulmonary surfactants.

## Data Availability

The datasets generated and analyzed during the current study are available from the corresponding author on reasonable request.

## Supplementary Materials

The following supporting information can be downloaded at: https://www.mdpi.com/article/10.3390/ma15186353/s1, Figure S1: The surface pressure–area isotherms of mixed monolayers of LM and ADON or SDON measured at 36.6 °C. Figure S2: The surface pressure–area isotherms of monolayers of LM and ectoine in presence of ADON or SDON measured at 36.6 °C. Figure S3: The surface pressure–area isotherms of monolayers of LM and budesonide drug in presence of ADON or SDON measured at 36.6 °C. Figure S4: The time-dependent decrease in surface tension curve of MPS at constant interfacial area (at 36.6 °C).
