# Supplementary material for: Interactions between O2 Nanobubbles and the Pulmonary Surfactant in the Presence of Inhalation Medicines
Source: Materials (Basel). 2022 Sep 13;15(18):6353. doi: 10.3390/ma15186353 (PMC9503299; doi:10.3390/ma15186353)
Supplement: Supplementary file 1 [file materials-15-06353-s001.zip › materials-1865282-supplementary.pdf]

Supplementary Materials

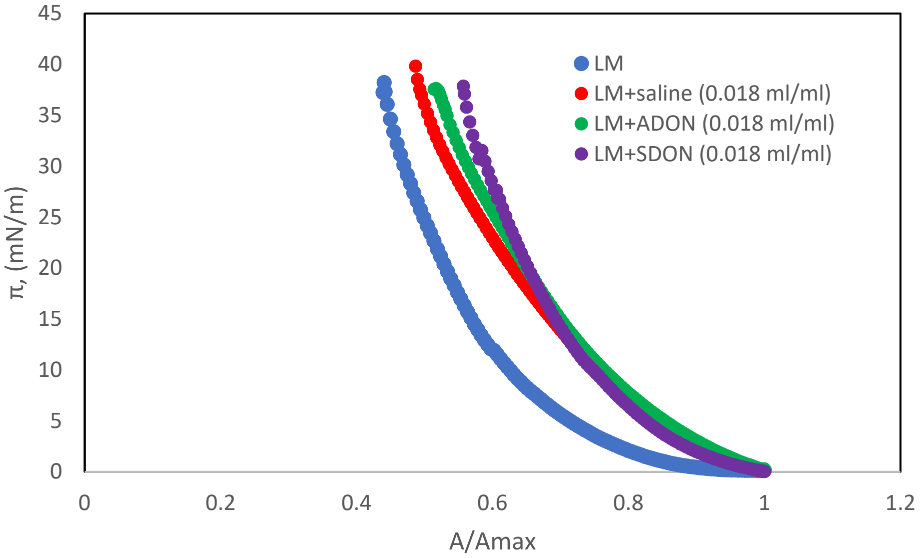

Figure S1: The surface pressure–area isotherms of mixed monolayers of LM and ADON or SDON measured at 36.6°C.

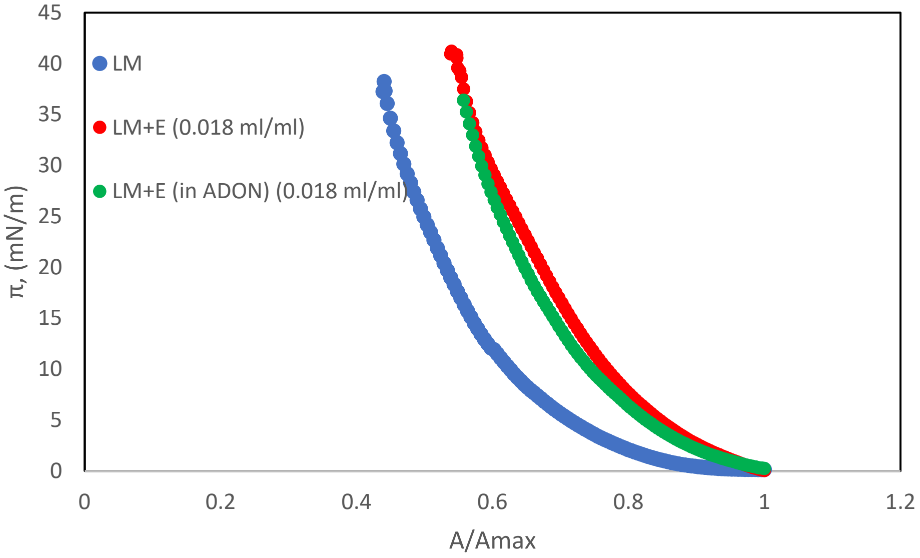

Figure S2: The surface pressure–area isotherms of monolayers of LM and ectoine in presence of ADON or SDON measured at 36.6°C.

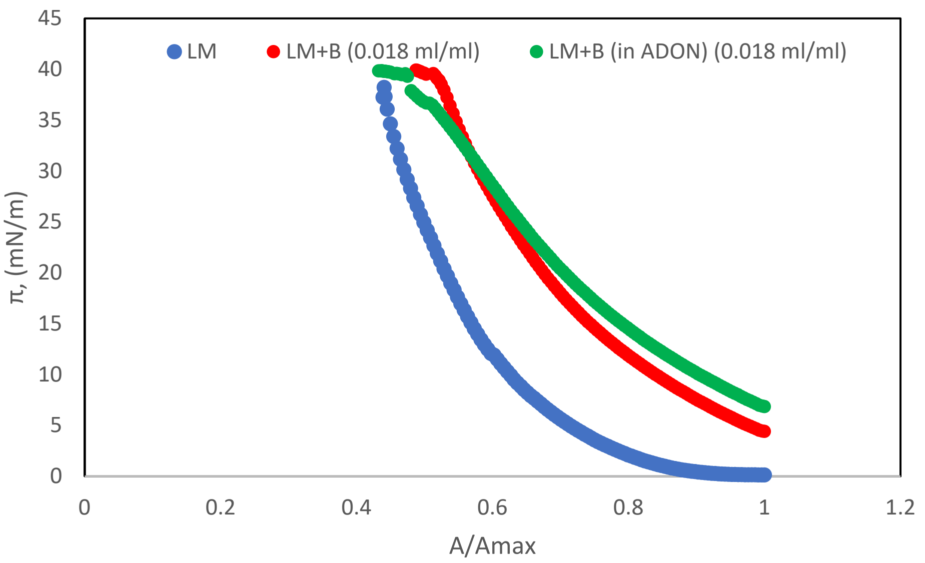

Figure S3: The surface pressure–area isotherms of monolayers of LM and budesonide drug in presence of ADON or SDON measured at 36.6°C.

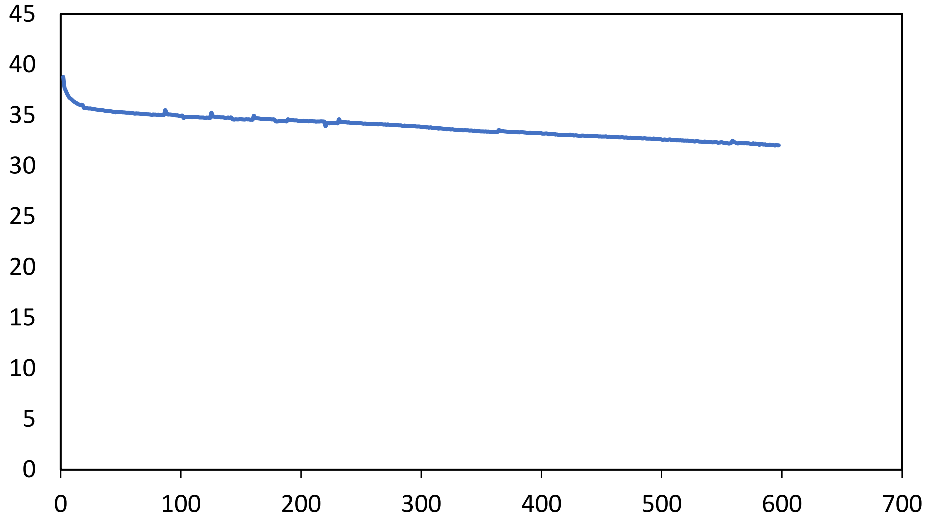

Figure S4: The time-dependent decrease in surface tension curve of MPS at constant interfacial area (at 36.6°C).
